# Signs and symptoms associated with primary tooth eruption: a clinical trial of nonpharmacological remedies

**DOI:** 10.1186/s12903-015-0070-2

**Published:** 2015-07-28

**Authors:** Mahtab Memarpour, Elham Soltanimehr, Taherh Eskandarian

**Affiliations:** Prevention of Oral and Dental Disease Research Center, School of Dentistry, Shiraz University of Medical Sciences, Shiraz, Iran; Department of Pediatric Dentistry, School of Dentistry, Shiraz University of Medical Sciences, Shiraz, Iran

**Keywords:** Teething, Primary teeth, Remedy, Tooth eruption, Symptoms

## Abstract

**Background:**

To evaluate disturbances in primary tooth eruption and their management with nonpharmacological remedies.

**Methods:**

In this nonrandomized clinical trial, 270 children aged between 8 and 36 months were selected and divided into 5 groups with 54 children initially enrolled in each group. The children were seen during an 8-day period during tooth eruption. At each appointment data were recorded from oral examination, tympanic temperature measurement and a questionnaire. The five methods used as remedies to reduce teething symptoms were: 1) cuddle therapy, 2) ice, 3) rubbing the gums, 4) teething rings and 5) food for chewing. Teething symptoms, the type of erupted tooth, symptoms of recovery and the mother’s satisfaction with treatment were evaluated.

**Results:**

Two hundred and fifty four children (mean age 16 ± 7.2 months) completed the study. The most frequent teething symptoms were drooling (92 %), sleep disturbances (82.3 %) and irritability (75.6 %). These symptoms were more pronounced in low birth weight children (*p* > 0.05). Canine eruption led to more loss of appetite than incisor (*p* = 0.033) or molars eruption (*p* = 0.014). Low grade increases in body temperature were observed only on the day of eruption (36.70 ± 0.39 °C), when body temperature was significantly different compared to the day before and the day after eruption (both *p* < 0.001). There was no significant correlation between fever as reported by mothers and temperature readings obtained by the investigators. The most favorable results for time to recovery and the mother’s satisfaction were seen when teething rings were used, followed by cuddle therapy and rubbing the gums.

**Conclusions:**

There was no association between teething and symptoms such as fever or diarrhea. Low birth weight children may have more teething symptoms. Teething rings, cuddle therapy and rubbing the gums were the most effective methods to reduce symptoms.

**Trial registration:**

Iranian Registry of Clinical Trials: code IRCT201211127402N3

## Background

Teething is a normal physiologic process consisting of intraosseous tooth movement in the jaw until the tooth emerges in the oral cavity [1, 2]. Tooth eruption takes place during an 8-day window that includes 4 days before tooth eruption, the day of eruption and the 3 subsequent days [3]. Systemic and local signs and symptoms ascribed to primary tooth eruption include general irritability, sleep disturbances, crying, fussiness, rhinorrhea, facial flushing, fever, diarrhea, loss of appetite, drooling, ear rubbing on the side of the erupting tooth, inflammation of the gingiva overlying the tooth, gum irritation, and increased biting [3–8]. Epidemiological studies have reported different prevalences of disturbances during primary tooth eruption, ranging from 95 % according to Cunha et al. [9] to 68 % according to Noor-Mohammed and Basha [10].

Although teething may cause problems for children, there is controversy regarding the direct relationship between tooth eruption and systemic symptoms. Some studies have failed to find any causal relationship between teething and symptoms such as fever, diarrhea, rashes or infections [1, 3, 4]. However, other research reported that parents and health care personnel do perceive some associations [8, 11, 12].

Over the years several methods based on popular and traditional beliefs and folk practices have been used to relieve teething symptoms. In some cultures aggressive, potentially harmful methods have been used such as local blistering, cautery [7] or gum lancing [13] for erupting teeth. Although some methods have been assumed to be safe and easy to use, such as teething necklaces or quack remedies, they increase the risk of strangulation or aspiration of small beads [14]. Other approaches involve the use of opiates, poisons such as lead acetate, mercury and bromide [7], or cooling baths to treat fever [15].

Some dentists may recommend using teething gels that may contain benzocaine or choline salicylate to reduce pain. These chemical products should be used carefully due to the risk of methemoglobinemia, interference with the gag reflex (and subsequent choking) [3, 16] and intoxication [17]. In other words, pharmacological products such as topical analgesics or systemic medications may lead to complications or have side effects [6, 12, 18]. Some parents prefer to use safer nonpharmacological methods as remedies for teething problems, such as homeopathic and natural remedies [3], behavioral therapy, chewing clean, cool objects such as a chilled teething ring or rattle, chilled hard vegetables or gingival massage with a cold, wet washcloth [12, 19–21]

Because of the controversy regarding the seriousness of some disturbances during tooth eruption, this study was designed to evaluate teething signs and symptoms in children younger than 3 years of age. An additional aim was to compare the clinical effectiveness of five nonpharmacological methods used as remedies to relieve teething disturbances. To our knowledge no clinical studies to date have been designed to compare the effectiveness of these methods in very young children.

## Methods

The research protocol was approved by the Human Ethics Review Committee of the Faculty of Dentistry, Shiraz University of Medical Sciences. For this 5-month, nonrandomized clinical trial, which started in June 2013, 270 noninstitutionalized children (i.e., receiving care at home) were enrolled at three local public health care centers in Shiraz.

Inclusion criteria: The children were between 8 and 36 months of age. All had at least one erupted primary tooth (no natal or neonatal teeth), and the parents were familiar with teething symptoms. The children also had at least one primary tooth in the process of eruption [22].

Exclusion criteria: The exclusion criteria included history of medical treatment for any systemic disease that might influence the signs and symptoms of teething, current drug treatment, congenital physical or mental disability, oral or dental anomalies or disabilities, and lack of parental consent to participate in the research.

### Data recording

All parents were interviewed and informed about the aims of the study and the methods to be used, and all provided their informed consent in writing. One trained dentist (E.S.) was responsible for data collection from three sources: 1) interview with the parents and information recorded on a questionnaire completed by the researcher at each appointment, 2) tympanic temperature taken by the dentist, and 3) clinical examination by the dentist.

To ensure that all data were recorded correctly, first the dentist was given instructions by a senior author (M.M.) on how to perform the oral examination, interview the mothers and record teething symptoms in 25 children (pilot test). The dentist was also taught how to record body temperature with a tympanic thermometer. In the pilot test with a group of 25 mothers, all questionnaire items were clearly understood by all participants.

### Teething signs and symptoms

At the first appointment the child’s mother was asked about new erupting teeth if signs of tooth eruption had appeared, and data were recorded for each child with the help of a questionnaire designed on the basis of a comprehensive literature review. The first part of the questionnaire recorded demographic information about the child including age, gender, normal or low birth weight (<2500 g), general health status and dental history. Information was also noted about the mother’s level of education, employment and age. The questionnaire contained 27 items about local and systemic teething disturbances attributable to eruption. Four dentists specialized in pediatric dentistry evaluated the questionnaire, which was revised as necessary based on their comments. The children were allocated into five equal groups to receive a different nonpharmacological treatment as a teething remedy. The parents were asked to attend regular follow-up appointments with their child during 8 days.

### Oral examination

An initial oral examination was done before the tooth erupted; then the children were selected. During the 8-day window for tooth eruption [8], all data were collected during the 4 days before eruption, on the day of eruption and 3 days after eruption. The mothers were asked to come to the health clinic as soon as they observed the initial signs of tooth eruption. Then they were interviewed to record the occurrence symptoms during the previous 24 hours and the daily data record sheet was completed, including nonpharmacological treatments used as teething remedies. For oral examination the gingiva surfaces were cleaned by wiping with a cotton roll. Intraoral examination was done with a head light and palpation of the alveolar ridge with the index finger to palpate the incisal edge or tip of the tooth cusp. The day of tooth eruption was considered the day when the crown edge of the tooth had visibly emerged in the oral cavity and was no longer than 3 mm [22]. The type of erupting tooth (incisor, canine or molar) was also recorded. Body temperature was measured with a tympanic thermometer (MT 50, Microlife, Basel, Switzerland) at every appointment.

### Experimental groups

Five different methods were compared as teething remedies, with 54 children initially enrolled in each group.

1. Cuddle therapy based on child behavior therapy. This included extra attention, care and reassurance by parents. The participating mothers were advised to hug or cuddle the child when the child felt distressed or manifested discomfort because of teething symptoms. Activities to distract the child such as reading, singing or playing were also used [7, 23].
2. Pieces of ice wrapped in a towel or other soft cloth were placed on the gums and mucous membrane overlying the erupting teeth for 1–2 min, and this was repeated as necessary when the child manifested teething symptoms [3].
3. Rubbing the child’s gums: Mothers were instructed to apply a light massage with their clean fingertips or a very soft finger toothbrush for 1–2 min [3, 7].
4. Teething rings: A solid plastic teething ring (Panberiz, Bushehr, Iran) was given to mothers, who were asked to give the ring to the child to chew or bite on. The teething ring we used did not cause cavities or choking, and had advantages over liquid-filled rings [3, 7].
5. Food for chewing: The children in this group were selected among those who had started to eat solid foods. The mothers were instructed to give the child small pieces of a frozen fruit or vegetable such as banana, apple or cucumber to bite or chew under the mothers’ supervision to prevent swallowing chunks of food material [3, 7].

The next appointment was scheduled for each child after initial signs of tooth eruption were observed by the mothers and the researcher. All children were examined daily (between 9:00 and 12:00 AM) and the data were recorded for teething symptoms, body temperature [4], and recovery following use of the remedy. We defined a variable for recovery from different symptoms on the day of eruption and after eruption, and compared the results to the period before eruption. For example, if the child had a symptom during the 4 days before eruption and the symptom disappeared in subsequent follow-up appointments, the child was considered to have recovered from the symptom. If the symptom did not disappear or if it became worse, it was recorded as no recovery. Children who had no symptoms during the study period were excluded from the analysis of recovery. On the last day, all mothers were asked to rate their satisfaction with the remedy on a 4-point Likert scale from 1 (completely effective) to 4 (completely ineffective).

If the mothers did not follow the instructions or used other methods or medical treatment as remedies for teething problems, the child was excluded from the analysis. If systemic symptoms such as fever (temperature higher than 38 °C) [24], nausea, diarrhea or seizures were observed, the child was referred to a pediatrician [4]. The primary outcomes were clinical manifestations of tooth eruption, fever and recovery after the intervention. The mothers’ satisfaction was considered as a secondary outcome.

### Statistical analysis

All data are reported here as frequencies (percentages) and mean ± standard deviation. Demographic variables were compared between groups with one-way analysis of variance (ANOVA) and chi-squared tests. The associations between different teething symptoms and birth weight categories (normal vs. low birth weight) were determined with the chi-squared test. Trends in body temperature during the study period were determined with repeated measures ANOVA (*p* < 0.05). The agreement between body temperature reported by mothers and recorded by the dentist was measured as the kappa coefficient. Chi-squared tests and Fisher’s exact test were used to evaluate recovery during the days of eruption and after eruption in comparison to before eruption. All analyses were done with SPSS v.16 software.

## Results

**Fig 1:**
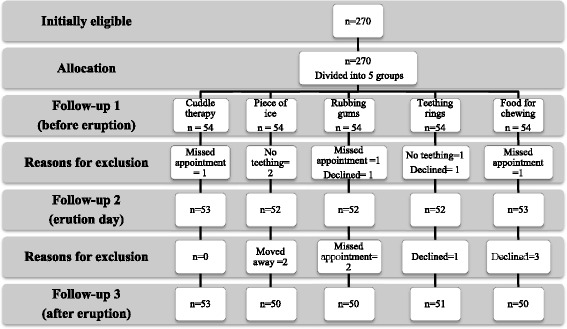
Flow diagram of study participants and research methodology

At baseline, 270 children between the ages of 8 and 36 months (mean age 16 ± 7.2 months, range 8.4 to 32.2 months) were enrolled. During follow-up, 16 children were excluded from the study due to missed appointments (n = 5), parents’ decision not to continue participating (n = 6), absence of tooth eruption (n = 3) or change in the place of residence (n = 2) (Fig. 1). The final sample consisted of 254 children who completed the study [128 (50.4 % female)]. There were no significant differences between the groups in sex ratio (*p* = 0.813) or mean age (*p* = 0.093). Mean age of the mothers was 30.95 ± 6.37 years. There was no significant difference between mothers in different groups in age (*p* = 0.121) or level of education (*p* = 0.735) (Table 1).

**Table 1:** Demographic information for children in each group

Variable		Group					*p* value
		1) Cuddle therapy	2) Piece of ice	3) Rubbing gums	4) Teething rings	5) Food for chewing	
Child age (months)		16 ± 5.24	17.92 ± 6.37	15.72 ± 5.07	16.08 ± 7.10	18.31 ± 6.11	0.093
Sex	Boy	29 (54.7 %)	25 (50 %)	24 (48 %)	22 (43.1 %)	26 (52 %)	0.813
	Girl	24 (45.3 %)	25 (50 %)	26 (52 %)	29 (56.9 %)	24 (48 %)	
Maternal age (year)		32.08 ± 6.56	29.42 ± 6.15	30.33 ± 6.84	30.51 ± 5.68	29.31 ± 4.57	0.121
Maternal education level	Less than diploma	9 (17 %)	10 (20 %)	12 (24 %)	11 (19.3 %)	8 (16 %)	0.735
	Diploma	26 (49.1 %)	28 (56 %)	28 (56 %)	26 (45.6 %)	25 (50 %)	
	University	18 (34 %)	12 (24 %)	10 (20 %)	20 (35.1 %)	17 (34 %)	

Quantitative characteristics were reported as the mean ± SD, and qualitative characteristics were reported and the frequency (%)

Most of the children [252 (99.2 %)] had one or more signs and symptoms during tooth eruption. Table 2 shows the mean frequencies of different teething disturbances during the study period. The most frequent teething symptoms were drooling (92 %), sleep disturbances (82.3 %) and irritability (75.6 %).

**Table 2:** Frequency (percentage) of clinical manifestations of tooth eruption in the study population

Clinical manifestation	Four days before eruption		Day of eruption		Three days after eruption	
Number (percentage)	n	%	n	%	n	%
Drooling	234	92.2	160	63	55	21.7
Diarrhea	62	24.4	29	11.4	1	0.4
Fever (Mother’s reports)	83	33.1	150	59.1	4	1.6
Lethargy	116	45.7	135	53.1	99	39
Loss of appetite	187	73.6	152	53.1	95	37.4
Lack of sleep	209	82.3	106	41.7	14	5.5
Gum irritation	175	68.9	88	34.6	7	2.8
Chewing objects	180	70.9	132	52	22	8.7
Finger sucking	67	26.4	40	15.7	25	9.8
Irritability	192	75.6	162	63.8	167	66.7
Red and inflamed gums	129	50.8	80	31.5	62	24.4
Gingival pain	103	40.6	86	33.9	36	14.2
Crying	190	74.8	112	44.1	12	4.7
Weight loss	214	84.3	110	43.3	106	41.7
Ear infection	9	3.5	4	1.6	0	0.0
Vomiting and nausea	24	9.4	24	9.4	22	8.7
Drooling + loss of appetite	187	73.6	213	83.9	118	46.5
Drooling + lack of sleep	209	82.3	180	70.9	63	24.8
No symptoms	1	0.4	1	0.4	2	0.8

The mean birth weight of the children was 2.91 ± 0.56 kg (range 1.50 to 4.20 kg). Children with low birth weight [n = 79 (33.6 %)] had more teething manifestations, and were 2.9 times as likely to develop diarrhea as normal birth weight children [OR = 2.90, CI 95 % (1.56-5.40), *p* = 0.001]. Low birth weight children (66 children, 83.5 %) were a 1.9 times as likely to have irritability as their normal birth weight counterparts [OR = 1.99, CI 95 %, (1.00-3.97), *p* = 0.047], and had more sleep disturbances (71 children, 89.9 %) than normal birth weight children [OR = 2.90, CI 95 % (1.00-5.241), *p* = 0.045]. There were no significant differences in other symptoms between children with low and normal birth weight (all *p* > 0.05).

**Fig 2:**
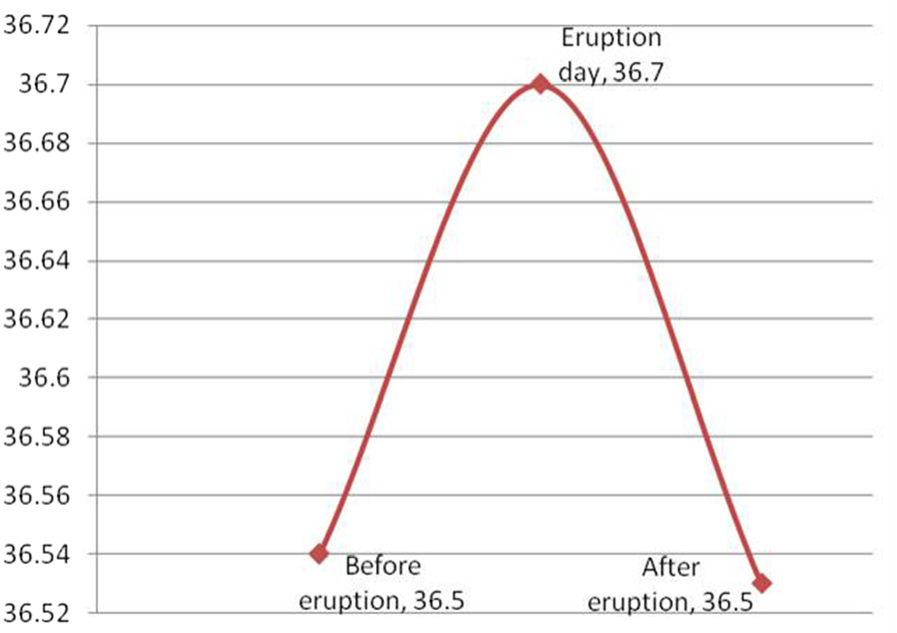
Mean tympanic temperature (degrees Celsius) 1) before eruption, 2) on the day of eruption and 3) after eruption

Figure 2 shows mean body temperature during the study period. On the day of eruption, mean body temperature (36.70 ± 0.39 °C) was 0.16 °C higher than before eruption, and 0.17 °C higher than after eruption. The difference was significant for both comparisons (*p* < 0.001). The difference between body temperature on the days before (36.54 ± 0.40 °C) and after eruption (36.53 ± 0.39 °C) was not significant (*p* = 0.601). There were considerable discrepancies between body temperature reported by the mothers and recorded by the dentist (Table 3).

**Table 3:** Agreement between fever reported by mothers and recorded by the dentist

	Fever		Kappa coefficient	*p* value
	Reported by mothers (n)	Recorded by clinician (n)		
Days before eruption	83	1	0.008	0.484
Day of eruption	150	3	0.011	0.239
Days after eruption	4	1	0.006	0.898

The kappa coefficient was used to calculate agreement between the two values

The frequencies of different types of erupted teeth were 131 incisors (51.5 %), 22 canines (8.7 %) and 101 molars (39.8 %). There were no significant differences in tooth eruption disturbances according to the type of tooth (*p* > 0.05). Only canines were associated with significantly more loss of appetite in comparison to incisors (*p* = 0.033) and molars (*p* = 0.014).

Table 4 shows the frequencies of recovery from different symptoms in the children who had teething disturbances as mentioned in the method part.

**Table 4:** Number (n) and percentage (%) of children in whom each treatment was effective for different symptoms

		Group					*p* value*
Clinical manifestation		1) Cuddle therapy (n = 53)	2) Piece of ice (n = 50)	3) Rubbing gums (n = 50)	4) Teething rings (n = 51)	5) Food for chewing (n = 50)	
Drooling	n %	47 (95.9)^a^	21 (44.7)^b^	29 (61.7)^b^	47 (94.0)^a^	37 (86.0)^a^	<0.001
Lethargy	n %	13 (59.1)^a^	10 (83.3)^a^	17 (70.8)^a^	11 (33.3)^b^	9 (36.0)^b^	0.003
Loss of appetite	n %	21 (52.5)^a^	13 (27.7)^b^	22 (50.0)^a^	17 (89.5)^a^	31 (83.8)^a^	<0.001
Lack of sleep	n %	41 (97.6)^a^	45 (97.8)^a^	41 (100)^a^	40 (93.0)^a^	32 (86.5)^b^	0.042
Gum irritation	n %	36 (100)^a^	42 (100)^a^	36 (94.7)^a^	29 (96.7)^a^	29 (100)^a^	0.27
Chewing objects	n %	29 (100)^a^	39 (92.9)^a^	39 (97.5)^a^	33 (78.6)^a^	21 (77.8)^b^	0.003
Finger sucking	n %	26 (89.7)^a^	40 (95.2)^a^	37 (92.5)^a^	40 (95.2)^a^	23 (85.2)^a^	0.525
Irritability	n %	6 (17.1)^a^	5 (10.9)^a^	6 (15.8)^a^	16 (42.1)^b^	16 (45.7)^b^	<0.001
Red and inflamed gum	n %	11 (64.7)^a^	26 (96.3)^b^	29 (90.6)^b^	21 (72.4)^a^	15 (62.5)^a^	0.006
Gingival pain	n %	18 (100)^a^	29 (96.7)^a^	16 (69.6)^b^	10 (76.9)^b^	14 (73.7)^b^	0.012
Crying	n %	41 (97.6)^a^	35 (89.7)^a^	38 (100)^a^	34 (91.9)^a^	32 (94.1)^a^	0.250

* Chi-squared test

In each row, different letters indicate significant differences between groups (Fisher’s exact test)

Table 5 shows the frequency of mothers’ reported level of effectiveness of each remedy. No side effects or unexpected effects were reported or observed in any group. There were significant differences in satisfaction between groups (*p* < 0.001), with the greatest effectiveness in group 4 (teething rings) and followed by groups 1 (cuddling) and 3 (rubbing the gums). The lowest levels of effectiveness were reported in group 2 (ice) and group 5 (food for chewing).

**Table 5:** Number and proportion of mothers who were satisfied with different remedies

Group	Completely effective		Moderately effective		Slightly effective		Completely ineffective	
	n	%	n	%	n	%	n	%
1) Cuddling	22	41.5	16	30.2	15	28.3	0	0.0
2) Pieces of ice	11	22.9	10	20.8	27	56.3	0	0.0
3) Rubbing gums	20	40.8	19	38.8	10	20.4	0	0.0
4) Teething rings	29	56.9	10	19.6	12	23.5	0	0.0
5) Food for chewing	10	20.0	15	30.0	25	50.0	0	0.0

## Discussion

Most of the children in the present study had one or more signs and symptoms during tooth eruption, in agreement with previous studies [9–11, 25]. However, the prevalences of each type of disturbance differed, possibly because of the influence of sample size, age, the method of data collection and the types of symptoms we studied [5, 6, 25]. As in previous studies, we found that tooth eruption was accompanied by local disturbances such as drooling and the urge to chew on objects [9, 11, 12, 26]. However, some reported that fever, diarrhea and vomiting were the symptoms most frequently related to tooth eruption [4, 7, 10, 19, 25, 27]. Like others, we found no relationship between systemic symptoms and teething [1, 3, 9, 11, 12, 20]. Tooth eruption is a physiological process and the manifestations we studied here may be coincidental with teething rather than causal consequences [6]. Moreover, the reporting of symptoms during tooth eruption may be influenced by healthcare factors as well as by parents’ knowledge, perceptions and beliefs [1, 8, 11, 12, 19, 25, 28–30].

As in the present study, some earlier reports found drooling to be one of the most common teething manifestations [12, 26, 31]. Increased salivation may result from irritation of the gums [10]. Excess saliva may lead to coughing or gagging, which should not cause alarm except in children with other signs of flu. Also, drooling may cause chin rash when saliva contacts the skin around the mouth. Cleaning the child’s mouth and chin is recommended to prevent the rash [7, 31].

Most of the children in our study preferred biting objects to reduce gingival irritation. Pressure from the erupting teeth is relieved by counter pressure from biting [3, 23]. However, contamination of the objects or the child’s fingers is a factor that can cause diarrhea. In this connection, swallowing excess saliva [3] or the release of IL-1beta and IL-8 cytokines [32] have also been suggested to contribute to looser stools during teething.

Parents believe their child’s behavior changes during tooth eruption. Specifically, gum soreness and pain may lead to irritability [7, 21, 31]. This reaction in association with increased levels of interleukin (IL-1beta) may cause loss of appetite and weight loss [32]. In our study some mothers reported irritability and pain in their child; however, the reliability of their reports of pain was impossible to judge because young children cannot verbally explain their pain experiences. Therefore mothers interpreted their child’s behavior and gestures to indicate pain based on, for example, facial expressions which may reflect other forms of stress or distress [33]. Pain is also related to increased levels of inflammatory mediators such as cytokines in the gingival crevicular fluid and the stimulation of nociceptive receptors [21, 32].

As in the present study, some earlier reports found mild increases (albeit within the normal range) in body temperature during tooth eruption [4, 8, 32]. Our results showed low-grade increases in temperature especially on the day of eruption, but not actual fever [4]. We found that many mothers held the misconception that teething leads to fever, whereas actual fever was found in few of the children in our study. This may reflect parents’ erroneous belief in the relationship between teething and fever [26, 28, 31]. Actual fever may be due to developmental changes in the child such as decreased maternal immunity and increased susceptibility to infection [7, 10, 19]. In addition, the release of IL-1beta and tumor necrosis factor (TNF) alpha may be casual factors in fever and sleep disturbances [32]. We used tympanic temperature, which provide easy, rapid and more accurate readings than axillary temperature [34].

Our results showed that the type of erupted tooth did not influence teething disturbances. Only canine eruption led to significantly more loss of appetite in comparison to incisors and molars. This difference may be related to the child’s discomfort and pain. One study reported teething disturbances were more prominent for incisors [10]. Additional studies are needed to evaluate the disturbances that may be associated with the eruption of different types of teeth.

The parents of low birth weight children in our study reported more teething disturbances compared to normal birth weight children. This may be due to lower “immune competence and increased vulnerability to infectious diseases” in the former subgroup [35]. In addition, maternal anxiety in the interactions with their children [36] may influence parents’ tendency to overestimate teething disturbances in low birth weight children.

Two main methods – pharmacological and nonpharmacological – have been recommended as remedies during tooth eruption. We used nonpharmacological remedies because of the parents’ attitudes towards using remedies which do not threaten their child’s health [3, 23]. According to our results, some methods such as teething rings, cuddle therapy and rubbing the gums were more effective than others. However, none of the methods was completely effective in ameliorating all the teething problems we studied. Teething rings and rubbing the gums reduced gingival irritation and finger sucking in the present study. The pressure caused by biting teething rings or pacifiers [7, 19, 20, 30] and gingival massage may decrease pain by overwhelming the sensory receptors [7, 9, 12].

Biting or sucking cold or frozen objects including fruits, vegetables or other foods causes localized vasoconstriction and decreases inflammation; in addition, the pressure on the gums reduces pain [3, 7, 11, 12, 19]. However, these remedies should be used only for children who are able to eat solid foods. Also, foods that are very hard should not be used, to avoid pain caused by bruising the gum [23]. Moreover, parental supervision is needed to prevent choking on small pieces of food [3, 7, 19]. In the present study pieces of ice or frozen foods were not effective, probably because the mothers found them difficult to use and they were not well accepted by the children. These problems may have influenced the time to recovery and the comparatively low level of parents’ satisfaction.

Behavior therapy and cognitive management are safe methods to manage sleep disturbances and irritation in children [23]. Our results showed that cuddle therapy was effective in controlling sleep disturbances and crying. Child crying and restlessness may be related to separation anxiety or attention seeking [21]. Therefore parental attention and care can be effective in assuaging some symptoms. These methods focus on reducing the sensation of pain during activities such as playing with the child, which can distract the child from pain [11, 23].

A potential limitation of the current study is that some mothers may not have followed our instructions correctly, or may have reported signs and symptoms inaccurately. Their reporting may be influenced by their beliefs regarding popular knowledge about teething, as exemplified by their reports of fever when their child’s temperature was only very slightly increased. In addition, our limited geographic setting and limited sample size should be considered study limitations [10].

On the other hand, the main strength of our study was the questionnaire we developed on the basis of a comprehensive literature review [9–11, 22, 25]. In addition, we measured body temperature during tooth eruption, recorded a variety of other symptoms during the eruption of different types of tooth, and compared our findings in normal birth weight and low birth weight children. In addition, we used nonpharmacological methods to reduce teething symptoms.

## Conclusions

The current study found no association between tooth eruption and systemic symptoms such as fever and diarrhea. However, mothers inaccurately reported slight increases in body temperature as fever. Low birth weight children had more teething manifestations than their normal birth weight counterparts. Use of a teething ring, cuddle therapy and rubbing the gums were the most effective methods to reduce symptoms.
